# Patient Characteristics Associated With Choosing a Telemedicine Visit vs Office Visit With the Same Primary Care Clinicians

**DOI:** 10.1001/jamanetworkopen.2020.5873

**Published:** 2020-06-17

**Authors:** Mary E. Reed, Jie Huang, Ilana Graetz, Catherine Lee, Emilie Muelly, Chris Kennedy, Eileen Kim

**Affiliations:** 1 Kaiser Permanente Division of Research, Oakland, California; 2 Rollins School of Public Health, Emory University, Atlanta, Georgia; 3 The Permanente Medical Group, Oakland, California; 4 University of California at Berkeley, Berkeley

## Abstract

**Importance:**

Video or telephone telemedicine can offer patients access to a clinician without arranging for transportation or spending time in a waiting room, but little is known about patient characteristics associated with choosing between telemedicine or office visits.

**Objective:**

To examine patient characteristics associated with choosing a telemedicine visit vs office visit with the same primary care clinicians.

**Design, Setting, and Participants:**

This cross-sectional study included data from 1 131 722 patients who scheduled a primary care appointment through the Kaiser Permanente Northern California patient portal between January 1, 2016, and May 31, 2018. All completed primary care appointments booked via the patient portal were identified. Only index visits without any other clinical visits within 7 days were included to define a relatively distinct patient-initiated care-seeking episode. Visits for routine physical, which are not telemedicine-eligible, were excluded. Data were analyzed from July 1, 2018, to December 31, 2019.

**Main Outcomes and Measures:**

Patient choice between an office, video, or telephone visit. Relative risk ratios (RRRs) for patient sociodemographic characteristics (age, sex, race/ethnicity, neighborhood socioeconomic status, language preference), technology access (neighborhood residential internet, mobile portal use), visiting the patient’s own personal primary care clinician, and in-person visit barriers (travel-time, parking, cost-sharing), associated with choice of video or telephone telemedicine (vs office visit).

**Results:**

Of 2 178 440 patient-scheduled primary care visits scheduled by 1 131 722 patients, 86% were scheduled as office visits and 14% as telemedicine visits, with 7% of the telemedicine visits by video. Choosing telemedicine was statistically significantly associated with patient sociodemographic characteristics. For example, patients aged 65 years and over were less likely than patients aged 18 to 44 years to choose telemedicine (RRR, 0.24; 95% CI, 0.22-0.26 for video visit; RRR 0.55; 95% CI, 0.54-0.57 for telephone visit). Choosing telemedicine was also statistically significantly associated with technology access (patients living in a neighborhood with high rates of residential internet access were more likely to choose a video visit than patients whose neighborhoods had low internet access: RRR, 1.10; 95% CI, 1.06-1.14); as well as in-person visit barriers (patients whose clinic had a paid parking structure were more likely to choose a telemedicine visit than patients whose facility had free parking: RRR, 1.70; 95% CI, 1.41-2.05 for video visit; and RRR, 1.73, 95% CI, 1.61-1.86 for telephone visit).

**Conclusions and Relevance:**

In this cross-sectional study, patients usually chose an in-person visit when scheduling an appointment online through the portal. Telemedicine may offer the potential to reach vulnerable patient groups and improve access for patients with transportation, parking, or cost barriers to clinic visits.

## Key Points

**Question:**

Which patient characteristics are associated with choosing either a telemedicine visit or an office visit with the same primary care clinician?

**Findings:**

In this cross-sectional study of data from 1.1 million patients with 2.2 million primary care visits, 14% of visits were scheduled as telemedicine (primarily by telephone), with patients more likely to choose telemedicine with their personal primary care clinician. Patient demographic characteristics, and office visit barriers were significantly associated with choosing telemedicine.

**Meaning:**

This study suggests a nuanced context for patient choice between a telemedicine visit and an office visit; the associations identified in this study may indicate opportunities for engagement with mobile technology access for those who face barriers to in-person visits.

## Teaser

This cross-sectional study examines patient sociodemographic characteristics, technology access, and potential in-person visit barriers to assess the association between patient characteristics and telemedicine vs in-person health care visits.

## Introduction

Telemedicine in various forms has the potential to engage patients through convenient health care access.^1,2^ Video or telephone visits can offer patients real-time access to a clinician without the need to go to a clinic. Patients in the US spend an average of 2 hours, including travel and wait time, for a 20-minute in-person office visit.^2,3^ Accordingly, while patient care-seeking for primary care has been associated with a wide range of financial and nonfinancial access barriers, patients who face greater barriers to in-person visits may choose a telemedicine visit if it offers greater convenience.^4,5,6^

While more than 1 million US patients have used telemedicine,^7^ direct-to-consumer services without in-person facilities often are not integrated with ongoing patient-clinician relationships or patient history within the electronic health record (EHR), which may create fragmentation from ongoing clinical care.^1,8,9,10,11,12,13^ Expanding technology use is increasing the availability of video telemedicine to patients, but limited consensus for payers remains a barrier.^14,15^ While video telemedicine has long been used in rural and specialty consultation, evidence remains particularly limited on video telemedicine use in primary care.^11,14,16,17,18,19,20^ To our knowledge, no prior research evidence from large studies of patient choice between a traditional office visit and a telemedicine telephone or video visit within ongoing patient-clinician relationships in community primary care has been performed.^1,19,21,22^

We examined an integrated delivery system setting in which patients using an online patient portal to schedule a primary care appointment were offered the choice to self-schedule a video telemedicine visit for primary care. Patients were offered a direct choice between 3 visit types with the same primary care clinician: office visit, video visit, or telephone visit. We examined rates of scheduled video or telephone telemedicine and patient characteristics associated with choosing telemedicine. We examined a broad set of patient characteristics including sociodemographic characteristics, technology access, and potential in-person visit barriers. We hypothesized that patient visit type preferences are associated with sociodemographic characteristics, and that greater technology access and barriers to in-person visits are associated with choosing telemedicine.

## Methods

### Setting

We examined primary care visits in Kaiser Permanente Northern California, a large integrated health care delivery system with more than 4 million members that uses a comprehensive outpatient-inpatient electronic health record (EHR; including outpatient, emergency, inpatient, laboratory, imaging, pharmacy history) and patient portal (website and mobile applications). The setting first implemented video visit technology in late 2014, with telephone visits used widely in clinical care since 2008.^23^ Plan members are linked with a personal primary care clinician but may also visit other primary care clinicians.

Starting in 2016, patients scheduling a primary care appointment through the patient portal must choose their visit type: office, video, or telephone visit (except for visits designated as a routine physical examination, which were offered only as office visits). Available clinicians included patients’ own personal primary care clinician (primarily MDs, including nurse practitioners) or other primary care clinicians the patient had visited recently. The schedule availability and clinicians available were comparable across visit types, with appointment availability generally within 3 days (often the same day). Telemedicine was generally exempt from any patient out-of-pocket cost-sharing. Only the small subset of patients with a high-deductible health plan defined by the US Internal Revenue Service for health savings account eligibility faced cost-sharing for telemedicine visits.

All primary care clinicians had access to technology to conduct patient-physician telephone or video visits through the existing clinician-facing EHR including from any office phone or computer or from a work-issued mobile device. As in an office visit, clinicians had full access to the patient’s EHR history and documented telemedicine visits directly within the EHR. Patients could receive a call for a telephone visit at any phone number and could access video visits from home or elsewhere in their daily lives directly through any internet-connected and video-enabled computer or mobile device. Use of video visits in this setting and initial patient experiences have been described previously.^4^

The Institutional Review Board of the Kaiser Foundation Research Institute approved the study protocol and materials and waived the requirement for written informed consent for participants in this data-only study. This study followed the Strengthening the Reporting of Observational Studies in Epidemiology (STROBE) reporting guidelines for cross-sectional studies.

### Study Population and Measures

We identified all completed primary care appointments booked via the patient portal from January 1, 2016, to May 31, 2018. We included only index visits without any other clinical visits within 7 days prior to define a relatively distinct patient-initiated care-seeking episode. We also excluded visits for routine physical examination, which are not telemedicine-eligible.

Our study examined several types of patient characteristics including patient sociodemographic characteristics and other measures of accessibility and affordability grouped into technology access, in-person visit barriers, and patient-clinician affiliation.^5^ For each patient included in the study, we used the EHR to identify patient sociodemographic characteristics (age, sex, race, language preference). Using the patients’ residential address, we defined their neighborhood socioeconomic status using 2010 US census measures at the census block group level, and neighborhood residential high-speed internet access level using FCC census tract level data. As additional measures of technology access, we captured patients’ prior mobile portal use as a measure of mobile device access, and video visit use in the past year to measure prior video visit experience. We extracted the patient’s insurance benefit cost-sharing for office visits, including copayment if a nondeductible plan, and deductible type if applicable (including health savings account–eligible high-deductible plans). We extracted the mean drive time from the patient’s residence to the nearest health system medical facility (61 total facilities), collected the type of parking offered at that facility and any parking fees (free parking lot or structure vs paid parking structure). We also used automated data sources to identify whether each visit was scheduled by a family care partner with permission to act for the patient through the patient portal, and whether the appointment was scheduled with the patient’s own personal primary care clinician.

### Statistical Analysis

We used multinomial logistic regression to examine the association (relative risk ratio [RRR]) between the chosen visit type (using office visit as the reference type) and patient characteristics, including patient sociodemographic characteristics (age, sex, race/ethnicity, neighborhood socioeconomic status, preferred language for health care), potential in-person visit barriers (out-of-pocket cost-sharing for office visits, drive time to clinic, facility parking garage and fee), technology access (neighborhood internet access level, portal access via mobile device in prior 365 days, video visits in prior 365 days), whether the appointment was booked by a care partner on behalf of the patient, and whether the clinician was the patient’s own personal primary care clinician. The multivariate model adjusted for patient medical problem (*International Statistical Classification of Diseases and Related Health Problems, Tenth Revision* code grouping of primary diagnosis), whether patient had preexisting chronic conditions (in clinical registry of asthma, heart failure, diabetes, and hypertension in the quarter prior to index visit) and medical center. Statistical significance was determined by 2-sided *P* < .05. Standard errors were adjusted for the repeated visits within patients using Stata, version 14.2 (StataCorp LLC).

## Results

**Figure 1.**
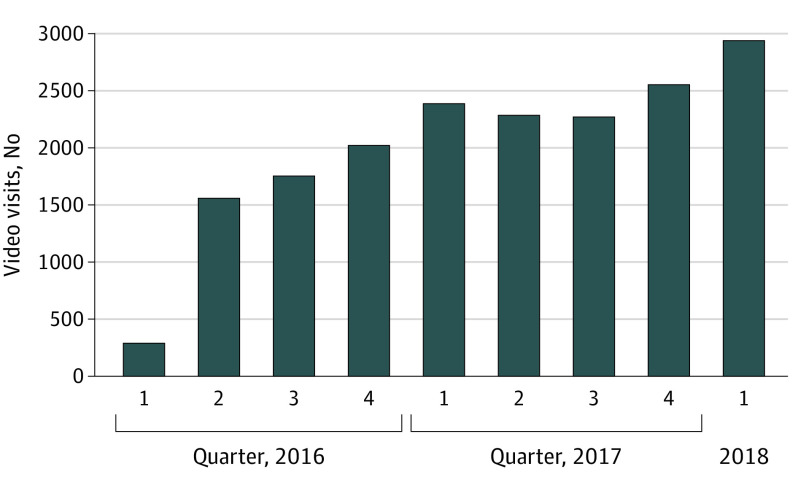
Patient-Scheduled Primary Care Video Visits, by Quarter Patient self-scheduling capability first added to the Kaiser Permanente Northern California patient portal in 2016.

A total of 2 178 440 eligible patient-scheduled primary care visits were scheduled by 1 131 722 patients. Among all patient-scheduled visits through the portal, 86% were scheduled as in-person office visits and 14% as telephone or video telemedicine visits. Figure 1 shows quarterly video visit counts during the study period which began when online video visit scheduling was first offered (telephone visits in eFigure in the Supplement). The Table shows demographic characteristics of patients scheduling a primary care visit included in the study.

**Table.** Patient-Scheduled Primary Care Visits and Patient Characteristics by Visit Type^a^

Characteristic	%			
	Total	Office visit	Video visit	Telephone visit
No.	2 178 440	1 870 552	20 115	287 773
Age, y				
<18	13.50	14.22	17.94	8.46
18 to <45	31.35	29.86	49.66	39.76
45 to <65	32.93	32.74	26.70	34.64
≥65	22.22	23.18	5.71	17.14
Male sex	45.14	46.09	43.21	39.08
Race/ethnicity				
White	59.76	60.04	49.25	58.64
Black	5.16	4.92	6.91	6.61
Hispanic	13.23	13.15	12.18	13.83
Asian	20.58	20.63	30.21	19.53
Other	1.27	1.25	1.46	1.39
Low neighborhood SES	15.58	15.42	15.21	16.60
English language speaker	95.07	94.97	95.68	95.66
Office visit cost-sharing, $				
0-10	21.66	21.87	15.57	20.76
15-30	46.02	46.16	47.15	45.02
35-75	5.68	5.75	2.68	5.40
Deductible	22.70	22.37	28.81	24.42
High deductible	3.24	3.17	5.09	3.55
Drive-time to facility, min				
≤20	79.38	79.69	77.00	77.53
20-30	11.16	11.06	11.21	11.80
>30	8.81	8.59	10.80	10.05
Facility parking				
Free	92.10	92.35	89.05	90.75
Paid garage	7.90	7.65	10.95	9.25
Neighborhood broadband internet access				
≤80%	64.11	64.12	58.48	64.43
>80%	35.54	35.52	40.90	35.26
Prior mobile portal use	58.36	56.83	63.83	67.87
Prior video visit experience	1.45	1.21	15.60	2.03
Appointment made by family member	14.28	14.87	17.97	10.21
Visit with patient’s personal clinician	87.91	87.76	90.02	88.73

Abbreviation: SES, socioeconomic status.

^a^ Study unit shown is patient-visit, percentages shown are column percentages. Neighborhood measures and drive time are based on patient’s residential address. Cost sharing is ordered generally by plan generosity, showing copayment if patient is enrolled in a plan with no deductible, and deductible type instead if the patient has a medical deductible. Prior mobile portal use and prior video visit use measure are based on prior 365 days.

### Sociodemographic Characteristics

**Figure 2.**
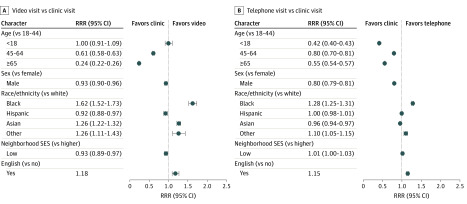
Relative Risk Ratios (RRRs) of Patient Sociodemographic Characteristics and Telemedicine Visit Choice A, Video visit vs clinic visit comparison. B, Telephone visit vs clinic visit comparison. SES indicates socioeconomic status.

Adjusted associations between patient sociodemographic characteristics and telemedicine visit choice are presented in Figure 2. After adjustment, female patients and patients aged 18 to 44 years were more likely to choose a telemedicine visit (either phone or video) than male patients or patients of other ages. For example, patients aged 65 and over were less likely than patients aged 18 to 44 to choose telemedicine (RRR, 0.24; 95% CI, 0.22-0.26 for video visit; RRR, 0.55; 95% CI, 0.54-0.57 for telephone visit).

Black patients were significantly more likely than white patients to choose both types of telemedicine, with higher RRRs for video visits (RRR, 1.62; 95% CI, 1.52-1.73 for video visit; RRR, 1.28; 95% CI, 1.25-1.31 for telephone visit), while Hispanic patients were only less likely than White patients to choose video (RRR, 0.92; 95% CI, 0.88-0.97), and Asian patients were more likely than white patients to choose video (RRR, 1.26; 95% CI, 1.22-1.32), but less likely than white patients to choose telephone (RRR, 0.96; 95% CI, 0.94-0.97). After adjustment, black patients were more likely to choose both phone and video visits than any other race/ethnicity.

Patients living in a lower-socioeconomic status neighborhood were significantly less likely to choose a video visit (RRR, 0.93; 95% CI, 0.89-0.97). Patients with documented non–English-language preference were significantly less likely to choose either type of telemedicine than English speakers.

### Barriers to In-Person Office Visits

**Figure 3.**
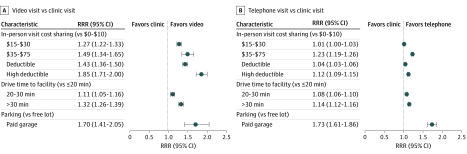
Relative Risk Ratios (RRRs) for Barriers to In-Person Visits and Telemedicine Visit Choice A, Video visit vs clinic visit comparison. B, Telephone visit vs clinic visit comparison.

Adjusted associations between barriers to in-person visits and telemedicine visit choice are presented in Figure 3. Patients whose insurance benefit plans required higher out-of-pocket cost-sharing for office visits (higher copayment if no deductible, or higher deductible if any deductible) were more likely to choose a telemedicine visit than patients with lower cost-sharing for office visits. For example, patients with an office visit copayment of $35 or more were significantly more likely to choose a video visit than patients with a $0-10 copayment (RRR, 1.49; 95% CI, 1.34-1.65) as were patients with a high-deductible plan (RRR, 1.85; 95% CI, 1.71-2.00).

Similarly, patients with longer relative driving time from home to the medical facility were significantly more likely to choose a telemedicine visit. Patients with a drive time longer than 30 minutes were significantly more likely than those with a less than or equal to 20-minute drive time to choose both video telemedicine (RRR, 1.32; 95% CI, 1.26-1.39) and telephone telemedicine (RRR, 1.14; 95% CI, 1.12-1.16). In addition, patients who may have needed to pay for parking in a garage structure were more likely to choose a telemedicine visit than patients whose facility had free parking (RRR, 1.70; 95% CI, 1.41-2.05 for video visit; RRR, 1.73; 95% CI, 1.61-1.86 for telephone visit).

### Technology Access and Personal Clinician

**Figure 4.**
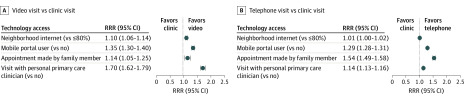
Relative Risk Ratios (RRRs) for Patient Technology Access and Personal Clinician With Telemedicine Visit Choice A, Video visit vs clinic visit comparison. B, Telephone visit vs clinic visit comparison.

Adjusted associations between patient technology access and personal clinician with telemedicine visit choice are presented in Figure 4. Patients living in a neighborhood with higher rates of residential internet access were more likely to choose a video visit than patients whose neighborhoods had lower internet access (RRR, 1.10; 95% CI 1.06-1.14), as were patients with mobile device access defined by recent history of accessing the patient portal by mobile device (RRR, 1.35; 95% CI, 1.30-1.40). Prior experience with a video visit within the past year was associated with telemedicine choice (RRR, 11.39; 95% CI, 10.82-11.99 for video visit; RRR, 1.53; 95% CI, 1.48-1.58 for telephone visit). Patients whose visit was scheduled by a family care partner on their behalf were more likely to have a telemedicine visit rather than an office visit (RRR, 1.14; 95% CI, 1.05-1.25 for video visit; RRR, 1.53; 95% CI, 1.49-1.58 for telephone visit).

Patients were also more likely to schedule a telemedicine visit if they were visiting their own personal primary care clinician than visiting another primary care clinician (RRR 1.70, 95% CI, 1.62-1.79 for video visit; RRR 1.14, 95% CI, 1.13-1.16 for telephone visit).

## Discussion

In this study, we found that patients scheduled 86% of visits as office visits and 14% as telemedicine visits, with 7% of the telemedicine visits scheduled as video visits. While most patients scheduling a visit through the patient portal chose a traditional office visit, we found that telemedicine preference varied by patient age and race, was supported by greater technology access, and appeared to represent a convenient option to access some health care visits when facing logistical barriers to an office visit including transportation time and visit costs. Patients were more likely to choose telemedicine with their own personal primary care clinician.

Our findings suggest that some patient groups may be particularly well-reached by telemedicine, including some vulnerable groups. Further, there appears to be nuance in choice between phone and video visit types. For example, black patients were more likely than white patients to choose both video and telephone telemedicine, however, Asian patients were less likely than white patients to choose telephone but more likely than white patients to choose video visit. Patients living in lower socioeconomic status neighborhoods were more likely to choose a telephone visit but were less likely to choose a video visit than patients in higher socioeconomic status neighborhoods. Because mobile devices are used in most video visits,^23^ and are increasingly the primary internet-access in vulnerable groups or those with lower health-engagement, mobile-friendly tools may represent valuable opportunities to engage these patients.^24^ Even though mobile devices are widely adopted, our findings of differences in visit choice by technology access indicate that technology access is not uniform.^25^

While early telemedicine efforts aimed to overcome travel barriers for specialty medical care access in remote locations, we found that patients were less likely to choose an office visit if the clinic was a farther drive or if parking was relatively more challenging.^26^ Also, because telemedicine was more likely chosen by family care partners helping patients to schedule appointments, those with additional transportation or mobility challenges may gain greater health care access through telemedicine. Telemedicine may also efficiently connect a family member to a live visit over geographic distances that might make attending a clinic visit together impractical.^27^ Further examination of specific patient groups whose needs may be particularly well met by telemedicine access is warranted.

Our findings suggest that patients with high out-of-pocket costs for office visits were more likely to choose a telemedicine visit. While most patients in our study had no out-of-pocket charge for telemedicine, in the small group with a high-deductible health plan that included an out-of-pocket charge for telemedicine, the cost-sharing differential still favored telemedicine. Notably, if visit cost-sharing was a factor in a patient delaying or avoiding any visit, these situations are not directly represented in our study sample.

Patients can face a wide range of financial and nonfinancial obstacles to receiving timely medical care, with barriers to primary care associated with more emergency department visits.^28^ We examined an integration of patient-initiated telemedicine visits with ongoing clinical care and patient-clinician relationships. While the landscape of telemedicine payment barriers is evolving, this study offers an opportunity to examine when patient-scheduled telemedicine is fully integrated into clinical care delivery and into the EHR. We offer specific findings about patient choice of telemedicine in the absence of differences in clinician and availability that may have consequences for direct-to-consumer telehealth. Our finding that patients were more likely to choose telemedicine with their own personal primary care clinician suggests that telemedicine might be especially preferred within ongoing patient-physician relationships. This telemedicine continuity may also hold clinical value as primary care continuity improves health care efficiency and health outcomes.^29^

### Limitations

This study has several limitations. Findings from this specific setting and limited to patient-initiated appointments scheduled online may not be generalizable to other less-integrated telemedicine delivery settings or different patient-initiated appointment-scheduling workflows. In deriving patient characteristics such as internet access, parking status, and socioeconomic status, we use general area characteristics and cannot directly associate any given barrier to an individual’s own experience. Another unmeasured common barrier, noted in our prior patient surveys, is a need to take time off from work to attend an in-person doctor’s office visit.^4^ In this study we do not have available automated data measures about patient work schedule and other personal responsibilities or visit barriers, but future research may examine this issue directly. While our analysis statistically adjusts for the general clinical area of a visit, it is still possible that patients are making visit-typed decisions based on perceptions of clinical need such as acuity, severity, or comorbidities that are not accounted for in our analysis. Future research may assess telemedicine follow-up visits within a given episode of care. Overall, our extensive set of patient and system variables allows for rigorous statistical adjustment, but an observational study cannot determine causation.

Our study period is limited to the initial implementation period after patient online-scheduled video telemedicine was first offered in the study setting. While telemedicine visits were primarily by telephone, video visit rates were increasing. We hypothesize that the telephone visit format was more familiar to patients in the study setting because telephone visits had been widely used in primary care for several years prior to the introduction of video visits. In the emerging area of telemedicine delivery of primary care, patient visit choice patterns will likely change over time and the study setting will also adapt its offerings. For example, more recently the study’s health system plans to integrate language interpretation services within telemedicine to improve access for non–English-speaking patients. Indeed, we found rates of choosing video were more than 10 times higher in patients who had prior scheduled video visit experience than in patients without prior experience. As patients and clinicians continue to gain experience with video telemedicine and the unique ways that it might fit particular personal and clinical situations, ongoing research on patient telemedicine preference and choice is needed. Also needed is further research examining quality and clinical outcomes associated with telemedicine visits and implementation factors that may affect adoption rates by both patients and clinicians.^26^

## Conclusions

While telemedicine can offer patients a convenient way to seek care from familiar clinicians, in this study of patient-scheduled primary care visits through a patient portal, we found that patients still primarily chose to schedule a traditional in-person office visit. Choosing a telemedicine visit was associated with patient sociodemographic characteristics, technology access and experience, in-person visit barriers, and continuity with the patient’s personal clinician, indicating a nuanced context within which patients may choose telemedicine rather than an office visit. While patient access to telemedicine visits may represent a transformative shift in patient-centered convenient health care access, the associations identified in this study of patient choice may indicate opportunities to engage non-white patients, patients with lower socioeconomic status, patients with mobile technology access, and those who face barriers to in-person visits.

## Notes

Supplement. **eFigure.** Number of Patient-Scheduled Primary Care Telephone Visits, by Quarter
