# Supplementary material for: Patient Characteristics Associated With Choosing a Telemedicine Visit vs Office Visit With the Same Primary Care Clinicians
Source: JAMA Netw Open. 2020 Jun 17;3(6):e205873. doi: 10.1001/jamanetworkopen.2020.5873 (PMC7301227; doi:10.1001/jamanetworkopen.2020.5873)
Supplement: Supplement. — eFigure. Number of Patient-Scheduled Primary Care Telephone Visits, by Quarter [file jamanetwopen-3-e205873-s001.pdf]

Supplementary Online Content

Reed ME, Huang J, Graetz I, et al. Patient characteristics associated with choosing a telemedicine visit vs office visit with the same primary care clinicians. *JAMA Netw Open*. 2020;3(6):e205873. doi:10.1001/jamanetworkopen.2020.5873

eFigure. Number of Patient-Scheduled Primary Care Telephone Visits, by Quarter

This supplementary material has been provided by the authors to give readers additional information about their work.

© 2020 Reed ME et al. *JAMA Network Open.*

Supplement

eFigure. Number of patient-scheduled primary care telephone visits, by quarter

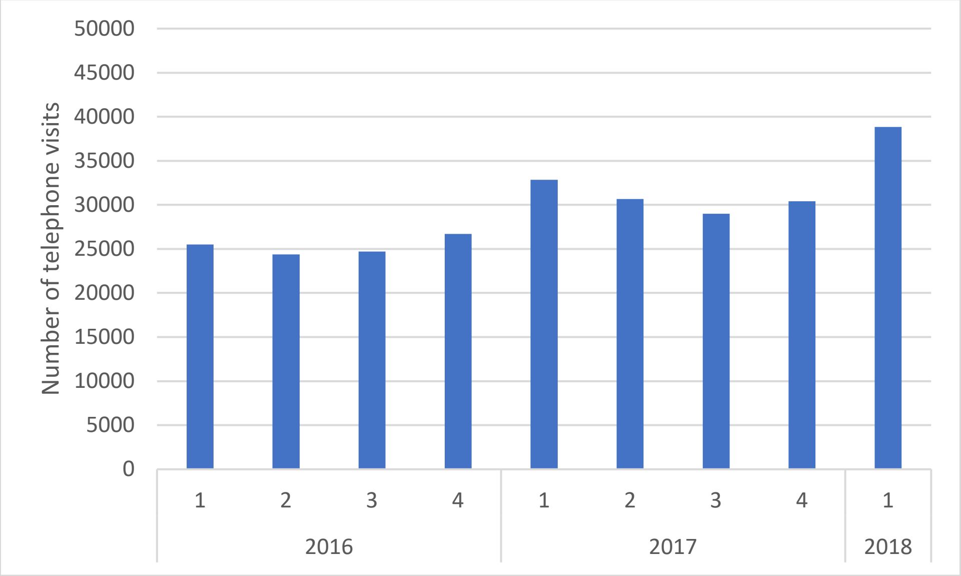

Note: Quarterly number of patient-scheduled primary care telephone visits.

© 2020 Reed ME et al. *JAMA Network Open.*
